# Sec-containing TrxR1 is essential for self-sufficiency of cells by control of glucose-derived H_2_O_2_

**DOI:** 10.1038/cddis.2014.209

**Published:** 2014-05-22

**Authors:** X Peng, P K Mandal, V O Kaminskyy, A Lindqvist, M Conrad, E S J Arnér

**Affiliations:** 1 Division of Biochemistry, Department of Medical Biochemistry and Biophysics, Karolinska Institutet, SE-171 77 Stockholm, Sweden; 2 Institute of Clinical Molecular Biology and Tumor Genetics, Helmholtz Zentrum München, German Research Center for Environmental health, Marchioninistr. 25, 81377 Munich, Germany; 3 Division of Toxicology, Institute of Environmental Medicine, Karolinska Institutet, SE-171 77 Stockholm, Sweden; 4 Department of Cell and Molecular Biology, Karolinska Institutet, SE-171 77 Stockholm, Sweden; 5 Helmholtz Zentrum München, Institute of Developmental Genetics, Ingolstädter Landstrasse 1, 85764 Neuherberg, Germany

**Keywords:** glucose, mouse embryonic fibroblasts (MEFs), reactive oxygen species (ROS), selenocysteine, thioredoxin reductase 1 (TrxR1)

## Abstract

It is commonly recognized that diabetic complications involve increased oxidative stress directly triggered by hyperglycemia. The most important cellular protective systems against such oxidative stress have yet remained unclear. Here we show that the selenoprotein thioredoxin reductase 1 (TrxR1), encoded by the *Txnrd1* gene, is an essential enzyme for such protection. Individually grown *Txnrd1* knockout (*Txnrd1*^−/−^) mouse embryonic fibroblasts (MEFs) underwent massive cell death directly linked to glucose-induced H_2_O_2_ production. This death and excessive H_2_O_2_ levels could be reverted by reconstituted expression of selenocysteine (Sec)-containing TrxR1, but not by expression of Sec-devoid variants of the enzyme. Our results show that Sec-containing TrxR1 is absolutely required for self-sufficient growth of MEFs under high-glucose conditions, owing to an essential importance of this enzyme for elimination of glucose-derived H_2_O_2_. To our knowledge, this is the first time a strict Sec-dependent function of TrxR1 has been identified as being essential for mammalian cells.

Reactive oxygen species (ROS) are generated as by-products of cellular metabolism,^1^ and thus increase in hyperglycemia.^2^ At physiological concentrations, ROS are regulators of transcription factor activities and serve as secondary messengers in intracellular signal transduction.^3^ However, excessive quantities of ROS, such as under hyperglycemic conditions, cause oxidative stress and cellular damage.^2^ Several antioxidant enzyme systems may however serve to protect cells and organisms from the toxic effects of excessive ROS. Among these, the thioredoxin (Trx)- and glutathione (GSH)-dependent systems together with specialized enzymes such as superoxide dismutases and catalase may act in concert.^4, 5^ Based upon the results of the present study, we suggest that the Trx system is absolutely required for protection against glucose-derived ROS, as shown using immortalized *Txnrd1* (gene encoding thioredoxin reductase 1) knockout (*Txnrd1*^−/−^) mouse embryonic fibroblasts (MEFs) as our model system.

Mammalian thioredoxin reductase 1 (TrxR1, encoded in mice by the *Txnrd1* gene) is a cytosolic selenoprotein with a selenocysteine (Sec, U) residue in a conserved C-terminal GCUG motif that is essential for its Trx-reducing activity.^6^ Using reducing equivalents from NADPH, TrxR1 supports a range of Trx-dependent antioxidant enzymes, such as peroxiredoxins (Prxs) and methionine sulfoxide reductases (Msrs). Prxs are important protective enzymes and also components of signaling cascades by modulating H_2_O_2_ levels,^7^ and Msrs repair oxidative damage on methionine residues of proteins.^8^ TrxR1 may also have other significant antioxidant functions through the reduction of a number of low-molecular-weight compounds as alternative substrates to Trx.^9^

Deletion of *Txnrd1* in mice yields early embryonic lethality.^10, 11^ Furthermore, conditional TrxR1 depletion in specific tissues of mice or its knockdown in cells can result in massive cerebellar hypoplasia,^12^ loss of self-sufficient growth under serum starvation,^13^ or abrogation of tumor formation in a xenograft model.^14^ However, there are also several observations showing that TrxR1 is not an essential enzyme in all types of cells and tissues,^11, 15, 16^ likely because of the fact that either chemical inhibition or genetic deletion of TrxR1 typically leads to Nrf2-activated upregulation of complementary GSH-dependent pathways.^17, 18^ Such findings also showed that TrxR1 is not absolutely required for support of DNA precursor synthesis through ribonuecleotide reductase (RNR), as long as GSH-dependent RNR support is maintained.^19^ In addition, many organisms have a closely related cysteine (Cys)-dependent non-selenoprotein TrxR, such as *D. melanogaster*, thereby illustrating that TrxR1 must not necessarily have a Sec residue for biological function.^20^ Mammalian TrxR1 is furthermore synthesized as a Cys-containing non-selenoprotein under selenium starvation conditions.^21, 22, 23^ These observations pose the question of whether there are any unique cellular functions of TrxR1 that can explain the lethality of its lack in mouse embryos, and whether there is any necessity of Sec *versus* Cys in TrxR1 in a cellular context.

Based upon the results of the present study, we conclude that Sec-dependent TrxR1 is absolutely required for protection of individually grown MEFs against glucose-generated H_2_O_2_. Interestingly, this protection against hyperglycemia-triggered oxidative stress could neither be sustained by increased levels of GSH and GSH-dependent enzymes in these cells nor by overexpression of a Sec-to-Cys-substituted variant of TrxR1.

## Results

### Verification of Txnrd1 status in MEF subclones

The MEF cell lines studied here include a parental MEF line that is functionally wild type with regard to TrxR1 status, having exon 15 of the *Txnrd1* gene flanked by flox sites (*Txnrd1*^*fl/fl*^), and the full knockout cell line that was clonally derived from *Txnrd1*^*fl/fl*^ cells after Cre treatment *in vitro* (hereafter referred to as *Txnrd1*^−/−^), as described previously.^16, 24^ The latter was used for subsequent transgenic expression of different N-terminally strep-FLAG tagged TrxR1 variants (SF-TrxR1), including overexpression of Sec-containing TrxR1 (*Txnrd1*^*498Sec*^), a Sec-to-Cys-substituted variant (*Txnrd1*^*U498C*^), Sec-to-Ser-substituted enzyme (*Txnrd1*^*U498S*^) and one variant truncated at the position of the Sec residue (*Txnrd1*^*498UAA*^).

**Figure 1:**
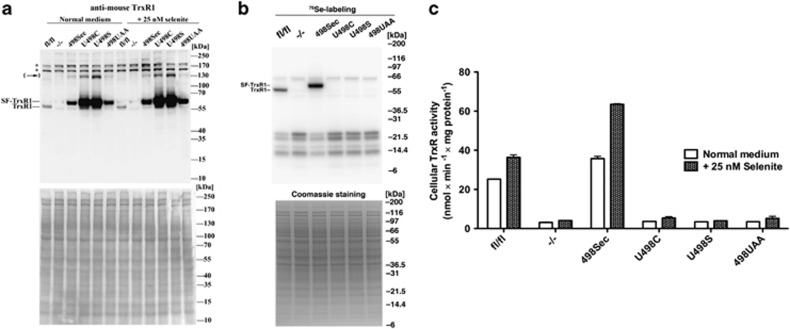
Characterization of expression levels, Sec incorporation and total cellular enzyme activity of TrxR in MEFs with depleted or reconstituted *Txnrd1* variants status. (**a**) Protein expression levels of TrxR1 incubated with or without 25 nM selenite supplementation in the medium for 24 h were analyzed by immunoblotting using reducing SDS-PAGE (top panel). Unspecific bands are indicated by asterisks (*) and TrxR1 dimeric bands are indicated by an arrow in parentheses. Endogenous (‘TrxR1') and reconstituted (‘SF-TrxR1') variants are indicated between the 55 and 70 kDa weight markers. Ponceau S staining was used as loading control (bottom panel). (**b**) Sec incorporation was determined using autoradiography of ^75^Se-labeled selenoproteins. The total proteins of lysed cells were analyzed on a reducing SDS-PAGE gel and exposed to a phosphor screen (top panel). Coomassie staining was used as loading control (bottom panel). (**c**) Total cellular TrxR activity was determined using a specific Trx-linked insulin disulfide reduction assay, with proteins of the same cell lysates as shown in (**a**) (*n*=2)

The *Txnrd1*^−/−^ cells and the expression of reconstituted TrxR1 variants were first confirmed as such by immunoblotting with antibodies against mouse TrxR1. This gave the expected results and furthermore showed that the reconstituted TrxR1 variants were overexpressed with regard to the endogenous TrxR1 level in the parental *Txnrd1*^*fl/fl*^ cells (Figure 1a). Autoradiography upon ^75^Se labeling of all cellular selenoproteins confirmed that Sec incorporation into the TrxR1 variants only occurred in the *Txnrd1*^*fl/fl*^ and *Txnrd1*^*498Sec*^ MEFs (Figure 1b). Quantification of total TrxR activity in the corresponding cell lysates revealed that only the *Txnrd1*^*fl/fl*^ and *Txnrd1*^*498Sec*^ MEFs expressed high enzymatic activity that was also responsive to selenium supplementation and ∼1.3- to 1.5-fold higher in the *Txnrd1*^*498Sec*^ cell line than in *Txnrd1*^*fl/fl*^ (Figure 1c).

### Compensatory upregulation of GSH systems in Txnrd1 knockout cells and their high dependence on GSH for viability

**Figure 2:**
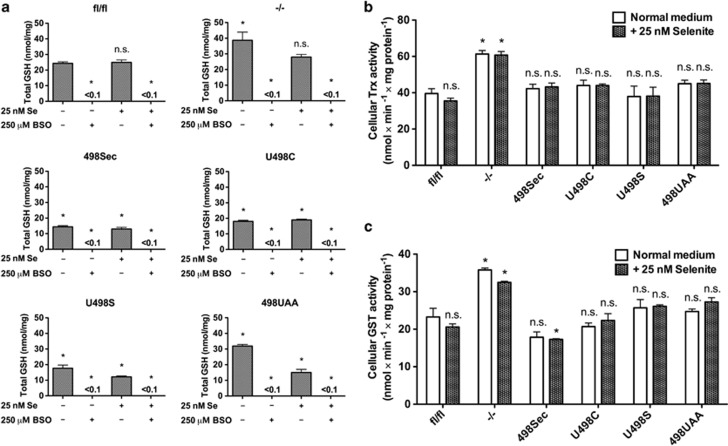
Compensatory changes in *Txnrd1*^−/−^ and reconstituted TrxR1 variant MEFs. (**a**) Total GSH contents of the MEF cell lines incubated with or without 250 *μ*M BSO and/or 25 nM selenite for 48 h are shown. (**b**) Total cellular Trx activities with or without 25 nM selenite supplementation were determined using cell lysates (*n*=3–4, ±S.E.M.). Significant differences between untreated *Txnrd1*^*fl/fl*^ without additional selenite and the other samples are indicated (*P<0.05; n.s., not significant, *P*>0.05). (**c**) Total cellular GST activities were determined using the same cell lysates in (**b**) (*n*=3–4, ±S.E.M.). Significant differences between untreated *Txnrd1*^*fl/fl*^ without additional selenite and the other samples are indicated (*P<0.05; n.s., not significant, *P*>0.05)

**Figure 3:**
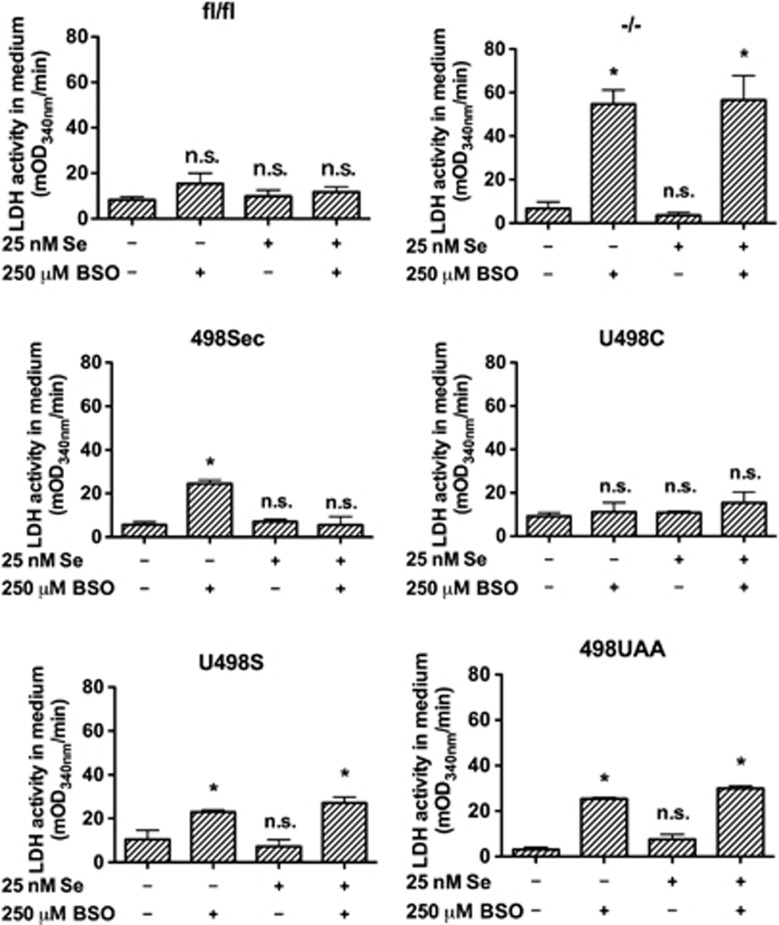
*Txnrd1* knockout cells are more sensitive to GSH depletion. The extent of cell lysis as indicator of cell death was estimated after 48 h of incubation with or without 25 nM selenite and/or 250 *μ*M BSO, by determining total LDH activity released to the extracellular medium (*n*=3, ±S.E.M.). Significant differences between the untreated cells without addition of selenite and the other samples within each cell line are indicated (*P<0.05; n.s., not significant, *P*>0.05)

**Figure 4:**
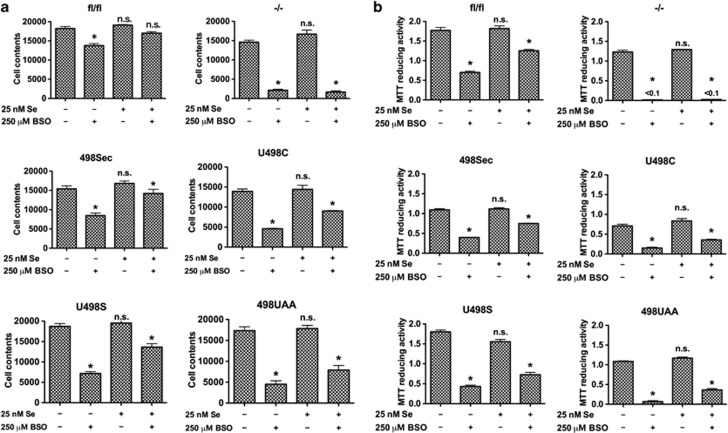
Growth of *Txnrd1-*impaired cells is diminished by GSH depletion. (**a**) Cell proliferation was determined by measuring total contents of cellular nucleic acids after 48 h of incubation, with or without 250 *μ*M BSO and/or 25 nM selenite (*n*=3, ±S.E.M.). Significant differences between the untreated cells without addition of selenite and the other samples within each cell line are indicated (*P<0.05; n.s., not significant, *P*>0.05). (**b**) Cell viability was also assessed through reduction of MTT (*n*=3, ±S.E.M.). Significant differences between the untreated cells without addition of selenite and the other samples within each cell line are indicated (*P<0.05; n.s., not significant, *P*>0.05)

Impairment of TrxR1 typically results in Nrf2 activation and upregulation of GSH-dependent enzymes.^10, 16, 25^ Here we found that only *Txnrd1*^−/−^, but not the other MEF lines, showed a significant elevation of Trx and glutathione transferase (GST) activities as well as total GSH (GSH plus GSSG) content compared with *Txnrd1*^*fl/fl*^ cells (Figure 2). In agreement with earlier findings,^16, 19, 24^ *Txnrd1*^−/−^ cells were found to be highly sensitive to GSH depletion by L-buthionine sulfoximine (BSO) treatment, as here illustrated by lactate dehydrogenase (LDH) leakage to medium, whereas no cytotoxicity with BSO was observed with the parental *Txnrd1*^*fl/fl*^ MEFs (Figure 3). Reconstituted expression of Sec-containing TrxR1 expression (*Txnrd1*^*498Sec*^) as well as the Sec-to-Cys mutant (*Txnrd1*^*U498C*^) rescued the cells from BSO-induced cytotoxicity (Figure 3) and restored cell growth (Figure 4). Intriguingly, reconstituted expression of the U498S or 498UAA variants in the knockout cells somewhat reverted the effects of TrxR1 deletion (Figures 3 and 4), without restoration of TrxR activity (Figure 1c), suggesting that expression of these proteins had some protective effects beyond that related to Trx reduction. Self-sufficient growth was, however, impaired in these cells (see next).

### Sec-containing TrxR1 is essential for self-sufficient growth of MEFs

**Figure 5:**
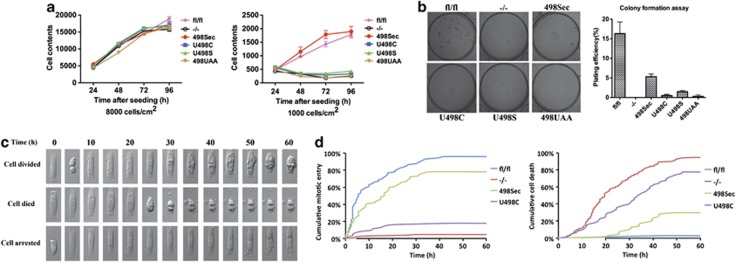
Sec-containing TrxR1 is essential for self-sufficient growth of MEFs. (**a**) Cells were seeded at densities of either 8000 cells/cm^2^ (left panel) or 1000 cells/cm^2^ (right panel) and cell proliferation was followed for 96 h by determination of total cellular nucleic acids in the culture wells (*n*=4, ±S.E.M.). (**b**) A total of 200 cells were seeded onto six-well plates and incubated for 7 days, whereafter colony formation capacity was assessed. A colony was defined as consisting of at least 20 cells. A representative plate is shown in the left panel, with a graph summarizing total plating efficiency in the right panel. Plating efficiency was calculated as the ratio of the number of colonies to the number of cells seeded (*n*=3, ±S.E.M.). (**c**) Single-cell cultures were maintained on fibronectin-coated micropatterns on glass cover slips and followed by time-lapse microscopy for 60 h. Examples of time-lapse montages for cells that proliferated (top), died (middle) or were arrested in growth (bottom) are illustrated. For full movies, see Supplementary Movies S1–S4. (**d**) The cumulative percentages of single cells that entered mitosis (left panel) or died (right panel) are plotted, as assessed from the single-cell cultures using time-lapse microscopy. Only very few cells displayed growth arrest (1%, 2%, 2% and 13% for *Txnrd1*^*fl/fl*^, *Txnrd1*^−/−^, *Txnrd1*^*498Sec*^ and Txnrd1^*U498C*^, respectively). At least 60 single cells were analyzed for each cell line

We found that an altered *Txnrd1* status had negligible effects on cell growth rates of MEFs when seeded in cultures at a higher density of 8000 cells/cm^2^. However, when seeded at the lower density of 1000 cells/cm^2^, only *Txnrd1*^*fl/fl*^ and *Txnrd1*^*498Sec*^ cells were able to proliferate, whereas the other cells completely failed to grow under such conditions (Figure 5a). These results suggested that self-sufficiency of the cells was affected. Indeed, colony formation assays showed that only *Txnrd1*^*fl/fl*^ and *Txnrd1*^*498Sec*^ cells survived at appreciable rates, suggesting that Sec-containing TrxR1 is necessary for growth of MEFs as single cells (Figure 5b). We therefore next used time-lapse microscopy to follow individual cells maintained on fibronectin-coated micropatterns, where the cells are devoid of cell–cell contacts but have similar cell–matrix contacts (Figure 5c and Supplementary Movies S1–S4). Almost all *Txnrd1*^*fl/fl*^ cells survived and entered mitosis at least once within 60 h after seeding, whereas only a few *Txnrd1*^−/−^ cells divided once and all of them died within this time frame (Figure 5d). Reconstitution with Sec-containing TrxR1, but not with the Sec-to-Cys mutant, provided significant rescue effects (Figure 5d). These findings suggested that Sec-containing TrxR1 has an essential role for self-sufficient growth of MEFs. Our further studies were therefore next focused on the molecular mechanisms that could explain the survival of *Txnrd1*^−/−^ cells in high-density cultures.

### Hydrogen peroxide removal rescues *Txnrd1*^ −/−^ MEFs

**Figure 6:**
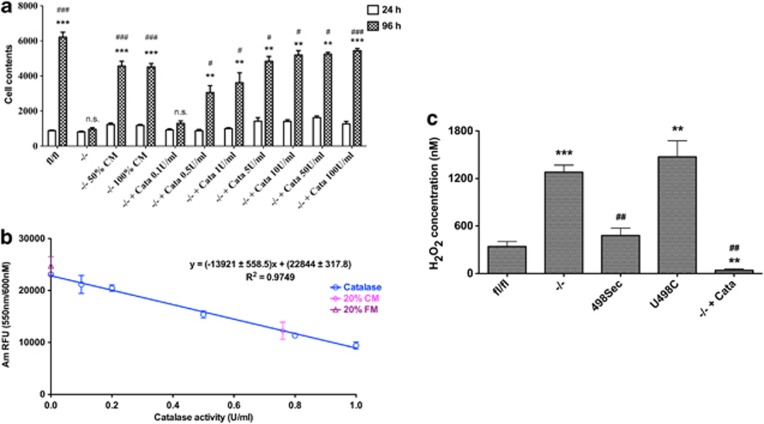
Removal of H_2_O_2_ rescues *Txnrd1*^−/−^ MEFs grown in sparse cell cultures. (**a**) Cells were seeded at a density of 1000 cells/cm^2^ with supplementation of the indicated amounts of conditioned medium (CM) from high-density cultures, or with catalase, and were cultured for either 24 or 96 h as indicated. Cell proliferation was then estimated by determination of total cellular nucleic acids (*n*=4–9, ±S.E.M.). Significant differences between the 96 h of *Txnrd1*^−/−^ and the other 96 h samples are indicated (*P<0.05; **P<0.01; ***P<0.001; n.s., not significant, *P*>0.05), and significant differences between the 24 h and 96 h of each treatment are also indicated (^#^*P*<0.05; ^##^*P*<0.01; ^###^*P*<0.001; n.s., not significant, *P*>0.05). (**b**) A standard curve with pure catalase was incubated with 10 *μ*M H_2_O_2_ (37 °C, 10 min), and the fluorescence signal indicating the remaining amount of H_2_O_2_ was determined using Amplex Red. The equation of the standard curve with *R*^2^ value is also shown. The corresponding catalase activities of 20% fresh medium (FM) or conditioned medium (CM) are also plotted onto the curve, as shown. According to this titration, 20% CM contained 0.76±0.12 units/ml catalase activity and 20% FM had no catalase activity (*n*=3–9, ±S.E.M.). (**c**) A total of 1.5 × 10^4^ *Txnrd1*^−/−^ cells, with or without supplementation with catalase (100 units/ml), or *Txnrd1*^*fl/fl*^, *Txnrd1*^*498Sec*^ and *Txnrd1*^*U498C*^ cells as indicated were seeded onto 96-well microtiter plates in HBSS buffer for 18 h, whereupon extracellular H_2_O_2_ was measured using Amplex Red (*n*=4–8, ±S.E.M.). Significant differences are indicated (*compared with *Txnrd1*^*fl/fl*^ MEFs, **P<0.01; ***P<0.001; ^#^compared with *Txnrd1*^−/−^ MEFs, ^##^*P*<0.01; ^###^*P*<0.001)

**Figure 7:**
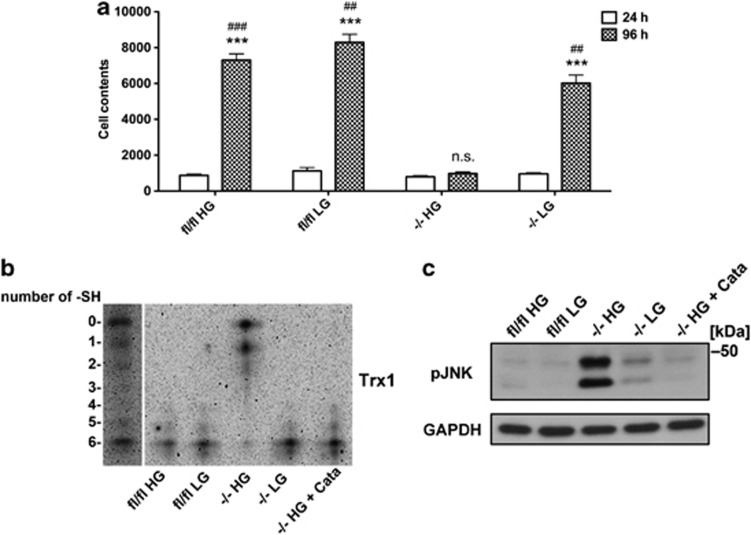
Low-glucose medium prevents cell death, Trx1 oxidation and JNK phosphorylation with *Txnrd1*^−/−^ MEFs grown in sparse cell cultures. (**a**) Cells were seeded at a low density of 1000 cells/cm^2^ in either high-glucose (25 mM glucose, HG) or low-glucose (5.5 mM, LG) medium, whereupon they were cultured for either 24 or 96 h. Cell proliferation was then estimated by determination of total cellular nucleic acids (*n*=6–9, ±S.E.M.). Significant differences between the 96 h of *Txnrd1*^−/−^ and the other 96 h samples are indicated (**P<0.01; ***P<0.001), and significant differences between the 24 and 96 h of each treatment are also indicated (^#^*P*<0.05; ^##^*P*<0.01; ^###^*P*<0.001; n.s., not significant, *P*>0.05). (**b**) *Txnrd1*^*fl/fl*^ or *Txnrd1*^−/−^ MEFs, with or without supplementation with catalase (100 units/ml), were cultured at 1000 cells/cm^2^ for 20 h in either HG (25 mM) or LG (5.5 mM) medium. The redox state of Trx1 was then analyzed by redox immunoblotting.^30^ The migration of Trx1 in a standard curve of MEF protein lysate treated so that Trx1 variants in all forms from 0 to 6 free Cys thiol groups being present^30^ is shown in the first lane. (**c**) The same set up as in (**b**) was used but phosphorylated JNK was determined by immunoblotting, using GAPDH as loading control

To investigate whether any secreted factor in conditioned medium (CM) of *Txnrd1*^−/−^ MEFs when cultured at high density (3.2 × 10^4^ cells/cm^2^, incubated for 24 h) could protect the cells, we collected such medium for use with *Txnrd1*^−/−^ MEFs seeded at a low density (1000 cells/cm^2^). Indeed, with either 50 or 100% CM, the *Txnrd1*^−/−^ MEFs could be rescued (Figure 6a). As catalase has previously been identified as a secreted survival factor for cells,^26, 27, 28^ we next supplemented fresh medium with pure catalase. As shown in Figure 7a, catalase supplementation to the medium was sufficient to rescue *Txnrd1*^−/−^ MEFs cultured at low density in a dose-dependent manner. We further assessed whether CM contained catalase activity and showed that 20% CM had 0.76±0.12 units/ml catalase activity, resulting in ∼4 units/ml catalase activity in full CM. Importantly, no catalase-like activity was found in fresh medium (Figure 6b). These findings collectively suggested that removal of H_2_O_2_ might be a key feature of the Sec-containing TrxR1 dependency in self-sufficient growth of MEFs.

We subsequently analyzed extracellular H_2_O_2_ levels in media of 200 *μ*l cell culture volumes upon 18 h of incubation. With this setup, we found that 1.5 × 10^4^ *Txnrd1*^*fl/fl*^ MEFs had generated an extracellular concentration of 340±64 nM H_2_O_2_. Notably, the medium from *Txnrd1*^−/−^ MEFs displayed an extracellular level of 1280±89 nM H_2_O_2_ that thus was increased fourfold compared with the parental MEFs. However, the levels were reverted by either reconstituted expression of Sec-containing TrxR1 (480±94 nM H_2_O_2_) or by addition of catalase (41±16 nM H_2_O_2_), but not by reconstitution of the Cys variant of the enzyme (1473±204 nM H_2_O_2_, Figure 6c), thereby correlating well with the protective effects of Sec-containing TrxR1 reconstitution or catalase supplementation (Figures 5d and 6a). These observations strongly supported the notion of an essential role of Sec-containing TrxR1 in cellular H_2_O_2_ elimination that could be compensated for by the presence of extracellular catalase activity.

### High glucose triggers oxidative stress-induced cell death of Txnrd1^ −/−^ MEFs grown in sparse cell cultures

All experiments above were performed in conventional MEF cell culture DMEM medium containing 4.5 g/l glucose (25 mM).^29^ To investigate whether the high glucose in this medium induced the oxidative stress-triggered cell death in *Txnrd1*^−/−^ MEFs, we next analyzed growth of the cells cultured at low density in DMEM medium using a lower glucose content (1 g/l glucose, 5.5 mM). Indeed, in sharp contrast to what was observed in the high-glucose medium, *Txnrd1*^−/−^ MEFs survived and proliferated in low-glucose medium (Figure 7a). As *Txnrd1* is depleted in *Txnrd1*^−/−^ cells and the redox status of its main substrate Trx1 is a marker of oxidative stress in the cytosol, we used a recently developed redox immunoblotting method^30^ to detect the number of free thiols in Trx1 of the MEFs cultured at low density in either high- or low-glucose medium. This revealed that only in *Txnrd1*^−/−^ MEFs cultured in high glucose, Trx1 was found mostly in its oxidized state, whereas this Trx1 oxidation was totally reversed by either growth in low glucose or by extracellular supplementation with catalase (Figure 7b). In parallel, we also determined the JNK phosphorylation state as a well-characterized marker for oxidative stress-related cell death.^31^ Indeed, a strong phosphorylation of JNK was observed in the *Txnrd1*^−/−^ MEFs grown in high-glucose medium, but not in the parental *Txnrd1*^*fl/fl*^ cells, in low-glucose cultures or upon catalase supplementation (Figure 7c).

Taken together, these findings demonstrated that the cell death observed in *Txnrd1*^−/−^ MEFs when grown at low density was induced by high glucose, high H_2_O_2_ and involved Trx1 oxidation and increased JNK phosphorylation. All these events could be totally prevented by either lowering the glucose content of the medium or by the presence of extracellular catalase activity, although neither of these events were seen upon growth in high glucose if the cells expressed Sec-containing TrxR1.

## Discussion

To our knowledge, this is the first study identifying a defined molecular function of Sec-containing TrxR1 that is essential for mammalian cells. With this function being protection against glucose-derived H_2_O_2_, several reflections can be made regarding the roles of selenium and parallel antioxidant systems in cells.

The massive cell death observed in *Txnrd1*-depleted MEFs when grown in sparse cell cultures, which could only be rescued by reconstituted expression of Sec-containing TrxR1, but not by expression of Sec-devoid variants of the enzyme including its Sec-to-Cys mutant, suggests that the GSH-dependent systems (upregulated in these cells) are not always redundant with the TrxR1-dependent antioxidant enzyme systems. Based upon our findings, it seems clear that the essential role of TrxR1 in these cells was indeed support of antioxidant protection, and not other roles such as support of DNA replication through RNR. The observation that extensive cell death was also prevented by CM from high-density cultures further supports that notion. In this case, catalase seemed to be an autocrine survival factor, and this is similar to previous findings with lymphocytes.^26, 27, 28^ An earlier study also found catalase secretion in MEF cultures.^32^ The findings also demonstrate that high H_2_O_2_ levels produced during oxidative stress may rapidly equilibrate between the intra- and extracellular space, which probably occurs through aquaporin-3,^33^ and explains how catalase present in the growth medium can protect cells from intracellularly produced H_2_O_2_.

It is worth noting that we found no evidence for increased Trx1 oxidation in the *Txnrd1*^−/−^ cells grown in low glucose or in high glucose upon addition of catalase. This shows that some additional cellular pathway, apart from TrxR1, can keep Trx1 in its reduced form. Recently, it was indeed shown that GSH and Grx1 can constitute such a backup system^34^ that may be further facilitated in *Txnrd1*^−/−^ cells because of their compensatory upregulation of GSH-dependent enzyme systems. Importantly, such alternative Trx1 reduction was evidently not sufficient in the *Txnrd1*^−/−^ MEFs for survival under increased oxidative stress as triggered by high glucose. It is here likely that TrxR1 is required, together with Trx, for sufficient support of Prxs to eliminate H_2_O_2_,^35^ but additional TrxR1-dependent antioxidant functions can also not be disregarded. It is important to note that Sec-containing TrxR1, but not the Sec-to-Cys version formed under selenium deficiency,^21, 22, 23^ could abolish the excessive amount of H_2_O_2_ release from *Txnrd1*^−/−^ cells and save them at low density in high glucose. This is indeed, to our knowledge, the first identification of a distinct molecular pathway where Sec-containing TrxR1 provides a unique and essential function in mammalian cells.

High glucose-induced production of ROS occurs mainly through two pathways:^36^ One is glucose oxidation in the tricarboxylic acid (TCA) cycle that yields superoxide and H_2_O_2_ through the mitochondrial electron transport chain;^36^ the other is the activation of protein kinase-C (PKC) that in turn activates NADPH oxidases.^36, 37^ Further studies are required to determine whether mainly one or both of these pathways are predominant sources of H_2_O_2_ in the *Txnrd1*^−/−^ cells.

The severe Trx1 oxidation and robust activation of JNK phosphorylation in the *Txnrd1*^−/−^ MEFs, with protection by catalase, support the notion that cell death can be directly triggered by H_2_O_2_, resulting in Trx1 oxidation, with subsequent activation of apoptosis signal-regulating kinase 1 (ASK1), JNK phosphorylation and finally cell death.^31, 38^ Recent results employing the same MEF cell lines as used in this study showed that *Txnrd1* depletion gave increased oxidation of PTP1B (protein tyrosine phosphatase 1B),^39^ that may also trigger signaling pathways with JNK phosphorylation. In addition to a direct signaling of apoptosis, necrosis can also contribute to oxidative stress-triggered cell death, because extensive ROS levels promote the opening of the mitochondrial permeability transition pore (MPTP) that can cause mitochondrial depolarization, ATP depletion and necrosis.^40^

Our findings may also explain why attempts to establish MEF cultures from homozygous *Txnrd1* knockout embryos at E8.5 invariably failed,^11^ whereas they could be established *in vitro* through treatment of *Txnrd1*^*fl/fl*^ MEFs with Tat-Cre recombinant protein.^16^ We thus speculate that cultured *Txnrd1*^*fl/fl*^ MEFs were able to reach a high density before depletion was performed, thereby yielding enough autocrine catalase activity to protect knockout cells from cell death. In contrast, isolation of *Txnrd1*^−/−^ MEFs directly from embryos results in low-density cultures that fail to survive. It is here worth emphasizing that clonal derivation of *Txnrd1*^−/−^ cells after Tat-Cre treatment was done in pyruvate-containing medium under low (5%) oxygen concentration^11, 15, 16, 24^ that may also yield lower oxidative stress because of direct scavenging of H_2_O_2_ by pyruvate,^41^ and less H_2_O_2_ formation in hypoxia.

In conclusion, here we found that elevated GSH-dependent enzyme systems were not sufficient to prevent oxidative stress-triggered cell death, and thus support of self-sufficient growth of *Txnrd1*^−/−^ MEFs, when grown in high-glucose MEF culture conditions. Sec-containing TrxR1, but not its Sec-to-Cys variant, was here exclusively required for cell survival and growth because of its critical role for H_2_O_2_ elimination.

## Materials and Methods

### Materials

Recombinant human wild-type Trx1 was generously provided by Arne Holmgren (Karolinska Institutet, Stockholm, Sweden). Recombinant rat TrxR1 (28.5 units/mg) was produced as described previously.^42^ Rabbit polyclonal anti-mouse TrxR1 antibody serum was a kind gift from Dr. Gary Merrill (Oregon State University, Corvallis, OR, USA). All other chemicals or reagents were obtained from Sigma-Aldrich Chemicals (St. Louis, MO, USA) unless stated otherwise.

### Isolation of mouse Txnrd1^−/−^ embryonic fibroblasts and reconstitution with variant forms of TrxR1

The establishment of *Txnrd1*^−/−^ MEFs has been described elsewhere.^16, 24^ In brief, cells with two loxP-flanked *Txnrd1* alleles were isolated from conditional *Txnrd1* knockout embryos,^11^ immortalized by cultivating them for at least 20 passages at low (5%) oxygen in Dulbecco's modified Eagle's medium (DMEM) with 4.5 g/l glucose content (25 mM glucose), 1 mM pyruvate and supplemented with 1% glutamine, 50 U/ml penicillin, 50 *μ*g/ml streptomycin (Invitrogen, Carlsbad, CA, USA) and 10% (v/v) fetal bovine serum (yielding∼15–20 nM selenium in final; PAA Laboratories, Cölbe, Germany).^43^ Tat-Cre fusion protein was applied to achieve *Txnrd1* depletion. Briefly, 5000/cm^2^ cells were incubated with 1 *μ*g Tat-Cre protein for 16 h in CD CHO medium (Invitrogen), and then the medium was exchanged for the DMEM medium mentioned above. After 24 h, the cells were washed, trypsinized and plated in limited dilution in 96-well plate. Cells were maintained at low (5%) oxygen with regular change of medium every third day. Outgrown clones were expanded and screened for deletion of *Txnrd1* by PCR and western blot,^24^ thus generating *Txnrd1*^−/−^ cell lines. To reconstitute TrxR1 expression in *Txnrd1*^−/−^ MEFs, a lentivirus-based approach was used to stably express murine wild-type TrxR1 and various mutants derived thereof in principle as described for glutathione peroxidase 4.^44^ Various mutant forms of TrxR1 were generated by site-directed mutagenesis against the Sec (Sec498) that was mutated to cysteine (*Txnrd1*^*U498C*^), serine (*Txnrd1*^*U498S*^) or truncated (*Txnrd1*^*498UAA*^), respectively, using the following sets of primers (mutated base pairs are in bold letter): Txnrd1_U498C_for: 5′-CTCCAGTCTGGCTGCTG**C**GGTTAAGCCCCAGT-3′, Txnrd1_U498C_rev: 5′-ACTGGGGCTTAACC**G**CAGCAGCCAGACTGGAG-3′, Txnrd1_U498S_for: 5′-CTCCAGTCTGGCTGCT**CC**GGTTAAGCCCCAGT-3′, Txnrd1_U498S_rev: 5′-ACTGGGGCTTAACC**GG**AGCAGCCAGACTGGAG-3′, Txnrd1_498UAA_for: 5′-TCCAGTCTGGCTGCT**AA**GGTTAAGC-3′, Txnrd1_498UAA_rev: 5′-GCTTAACC**TT**AGCAGCCAGACTGGA-3′. The reconstituted TrxR1 was furnished at the N-terminus with a tandem affinity purification enhanced tag (TAPe-tag) consisting of FLAG-2xStrep, and expressed with the natural Txnrd1-derived selenocysteine insertion sequence (SECIS) element in the 3′ UTR. *Txnrd1* knockout cells were transduced with lentiviruses encoding either wild-type or various mutants. 5 × 10^4^ cells were transduced with 10–15 *μ*l of virus supernatant. At 36 h post transduction, transduced cells were treated with 1 *μ*g/ml of puromycin for stable expression in batch cultures.

### Cell cultures

The established MEF variants were, unless indicated, cultured in DMEM with 4.5 g/l glucose content (25 mM glucose), no pyruvate and supplemented with 2 mM glutamine, 100 U/ml penicillin, 100 *μ*g/ml streptomycin (BioWhittaker, Lonza, NJ, USA) and 10% (v/v) fetal bovine serum (yielding ∼15–20 nM selenium in final; PAA Laboratories). DMEM with neither glucose nor pyruvate (Invitrogen) supplemented with 1 g/l glucose (5.5 mM) and the other reagents listed above was used for the low-glucose cultures. Cells were grown in humidified air containing 5% CO_2_ at 37 °C for all experiments.

### Preparation of cell lysates

All cell lines were seeded with or without 25 nM selenite supplementation 24 h before they were harvested and lysed in extraction buffer (50 mM Tris-HCl, pH 7.5, 2 mM EDTA, 0.5 mM phenylmethylsulfonyl fluoride and 0.5% Triton-X). The clarified supernatants after centrifugation (13 300 r.p.m., 15 min) were used to analyze either enzymatic activities or immunoblots. Total protein concentrations were determined with a Bradford reagent kit (Bio-Rad, Hercules, CA, USA).

### Immunoblot detection of TrxR1 isoforms

Total proteins were separated on a reducing SDS-PAGE gel and Ponceau S staining was used as loading control. A rabbit polyclonal anti-mouse TrxR1 primary antibody serum was used with the SuperSignal West Pico kit (Thermo Fisher Scientific, Waltham, MA, USA) according to the manufacturer's instructions, and the signals were detected utilizing a Bio-Rad ChemiDoc XRS scanner and the Quantity One 4.6.7 software.

### Enzyme activity assays

Cellular TrxR and Trx activities were determined using the previously described end-point insulin reduction assay.^45^ Cellular GST activity was determined based on enzyme-dependent conjugation of reduced glutathione with CDNB according to Habig *et al.*,^46^ as modified for a 96-well plate. Briefly, the assay consists of 2 mM GSH and 0.5 mM CDNB in phosphate buffer, and then the cell lysates were added and the reactions were monitored at 340 nm. Controls without proteins were treated as background.

### ^75^Se-radioisotope labeling

Cells were seeded and incubated with ^75^Se-labeled selenite (Research Reactor Center, University of Missouri, Columbia, MO, USA) in a final concentration of 1 *μ*Ci/ml for 48 h before they were collected and lysed. The clarified supernatants were fractionated on a reducing SDS-PAGE (Buffers, gel and equipment from Invitrogen). The gel was stained with Coomassie Blue to visualize total protein. After drying, it was then exposed on a phosphor screen and autoradiography was visualized with a Typhoon FLA 7000 (GE Healthcare Lifesciences, Little Chalfont, Buckinghamshire, UK).

### Cell viability assays

For analyses of viable cells, they were incubated with 0.5 mg/ml MTT (3-(4,5-dimethylthiazol-2-yl)-2,5-diphenyl-2H-tetrazolium bromide) at 37 °C for 4 h and thereupon dissolved in DMSO. Absorbance was measured at 550 nm, with cell-free samples as background.

### Cell proliferation and cytotoxicity assays

Cells were seeded for the indicated time points onto 96-well microtiter plates and subsequently treated as described. Cell proliferation was estimated based upon determination of total cellular nucleic acids, using the CyQUANT Cell Proliferation Assay (Life Technologies, Grand Island, NY, USA) according to the manufacturer's instructions. LDH efflux assays were used to assess cytotoxicity and were performed as described previously.^23^

### Quantification of total intracellular GSH and GSSG

Total intracellular GSH and GSSG concentrations were determined by the previously described glutathione reductase-DTNB recycling assay.^45^

### Colony formation assays

Cells was seeded into six-well plates, with subsequent growth for 7 days. On the day of analysis, colonies were fixed and stained with 6% glutaraldehyde containing 0.5% crystal violet, and then counted using an optical microscope. A colony was defined as consisting of at least 20 cells.

### Micropattern-based single-cell cultures and time-lapse imaging

In order to record single-cell growth, cells were plated on fibronectin-coated 80 × 15 *μ*m micropatterns on glass cover slips custom-produced by Cytoo (Grenoble, France). At 30 min after seeding, cover slips were washed to remove unattached cells, whereupon the slips were kept in a humidified atmosphere with the same high-glucose DMEM as in the other experiments covering the cells. Cells were imaged using differential interference contrast (DIC) at 37 °C in 5% CO_2_ on a Leica (Solms, Germany) DMI6000 imaging system, using a 20 × NA 0.4 objective. Images were acquired every 30 min for 60 h. The cumulative percentage of single cells that entered mitosis or died were plotted against incubation time, as described in the main text.

### Hydrogen peroxide assays

Levels of H_2_O_2_ were measured using Amplex Red as described previously.^47^ Briefly, 1.5 × 10^4^ cells were seeded into 96-well plates in 200 *μ*l of HBSS (Hank's Balanced Salt Solution, 1 g/l glucose) containing 20 *μ*M Amplex Red and 0.1 U/ml HRP, and were incubated at 37 °C for 18 h. Thereafter, fluorescence readings were recorded with excitation and emission wavelengths of 550 and 600 nm, respectively. Different concentrations of H_2_O_2_ (0–5 *μ*M) were used to generate a standard curve.

### Catalase activity assays

Indicated amounts of catalase (Sigma, St. Louis, MO, USA; catalog no. C1345) or medium samples were incubated with 10 *μ*M H_2_O_2_ at 37 °C for 10 min in HBSS in total volumes of 100 *μ*l in 96-well microtiter plates. For subsequent analyses of H_2_O_2_ levels, 100 *μ*l HBSS buffer containing 40 *μ*M Amplex Red and 0.2 U/ml HRPs was added to each well in 96-well microtiter plates and fluorescence readings were recorded immediately with excitation and emission wavelengths of 550 and 600 nm, respectively.

### Redox immunoblotting of Trx1

Redox immunoblotting of Trx1 was performed as described elsewhere.^30^

### Immunoblot detection of phosphorylated JNK

Immunoblot detection of the phosphorylated JNK was performed as described elsewhere.^48^

### Statistics

Values are presented as means±S.E.M. Statistical evaluation was performed with the Mann–Whitney test using the GraphPad Prism software, version 5.0 (GraphPad Software, San Diego, CA, USA). Asterisks or pounds signs denote statistically significant differences between the indicated groups of data (* or ^#^*P*<0.05; ** or ^##^*P*<0.01; *** or ^###^*P*<0.001).

## Abbreviations

Sec: selenocysteine
TrxR1: thioredoxin reductase 1
*Txnrd1*: gene encodes thioredoxin reductase 1
MEF: mouse embryonic fibroblast
ROS: reactive oxygen species
Trx: thioredoxin
GSH: glutathione
Prx: peroxiredoxins
Msr: methionine sulfoxide reductases
RNR: ribonuecleotide reductase
Cys: cysteine
GST: glutathione transferase
LDH: lactate dehydrogenase
BSO: L-buthionine sulfoximine
CM: conditioned medium
TCA: tricarboxylic acid
PKC: protein kinase-C
ASK1: signal-regulating kinase 1
PTP1B: protein tyrosine phosphatase 1B
MPTP: mitochondrial permeability transition pore
MTT: 3-(4,5-dimethylthiazol-2-yl)-2,5-diphenyl-2H-tetrazolium bromide
FBS: fetal bovine serum
CDNB: dinitrochlorobenzene
