# Supplementary material for: Sec-containing TrxR1 is essential for self-sufficiency of cells by control of glucose-derived H2O2
Source: Cell Death Dis. 2014 May 22;5(5):e1235–. doi: 10.1038/cddis.2014.209 (PMC4047868; doi:10.1038/cddis.2014.209)
Supplement: Supplementary Movie Legends [file cddis2014209x5.doc]

Supplementary information

Movie S1: Movie of micropattern-based single cell culture are made from images acquired every 30 min for 60 h for *Txnrd1^fl/fl^* MEFs in Figure 5c and d with frame rate 15 fps.

Movie S2: Movie of micropattern-based single cell culture are made from images acquired every 30 min for 60 h for *Txnrd1^-/-^* MEFs in Figure 5c and d with frame rate 15 fps.

Movie S3: Movie of micropattern-based single cell culture are made from images acquired every 30 min for 60 h for *Txnrd1^498Sec^* MEFs in Figure 5c and d with frame rate 15 fps.

Movie S4: Movie of micropattern-based single cell culture are made from images acquired every 30 min for 60 h for *Txnrd1^U498C^* MEFs in Figure 5c and d with frame rate 15 fps.
